# Effects of Increasing Glycerin Levels in Broiler Chickens

**DOI:** 10.3390/metabo14060308

**Published:** 2024-05-28

**Authors:** Elaine de Assis Carvalho, Weslane Justina da Silva, Denise Russi Rodrigues, Ludmilla Faria dos Santos, Camila Ferreira Rezende, Flávio Medeiros Vieites, Fabiana Ramos dos Santos, Fabiano Guimarães Silva, Cibele Silva Minafra

**Affiliations:** 1 Goiano Federal Institute of Education Science and Technology (F Goiano), Rio Verde 75901-970, GO, Brazil; blognenacarvalho@gmail.com (E.d.A.C.); vetludmilla@gmail.com (L.F.d.S.); camilagyn_88@hotmail.com (C.F.R.); fabiana.santos@ifgoiano.edu.br (F.R.d.S.); fabiano.silva@ifgoiano.edu.br (F.G.S.); 2 Samambaia Campus, University Federal of Goiás Program Postgraduate in Biotechnology and Biodiversity—PGBB/UFG, Goiânia 74001-240, GO, Brazil; weslanejds@gmail.com; 3 Ohio Agricultural Research and Development Center, The Ohio State University, 1680 Madson Ave, Wooster, OH 44691, USA; russirodrigues.1@osu.edu; 4 Department of Veterinary Medicine, Federal University of Juiz de Fora, Juiz de Fora 36036-900, MG, Brazil; fmvieites@yahoo.com.br

**Keywords:** corn replacement, glycerin, metabolism, organ biometrics, performance

## Abstract

Glycerin contributes to the animal’s energy metabolism as an important structural component of triglycerides and phospholipids. The present study was carried out to evaluate the effect of replacing corn with 0, 5, 10, and 15% of glycerin in terms of performance, digestibility, carcass yield, relative weights of gastrointestinal tract (GIT) organs, and nutrient metabolism. Four hundred chickens (40.0 g ± 0.05 g) were distributed in a completely randomized design with four treatments and five replicates. Growth parameters were measured at 7, 14, 21, and 42 days. Digestibility of crude protein and fat, carcass yield, relative weights of GIT organs, and biochemical blood profile were measured. The results were subject to an analysis of variance by Tukey’s HSD test (*p* > 0.05). The inclusion of 5%, 10%, or 15% of glycerin did not influence performance or affect the crude protein and fat digestibility in broilers (*p* > 0.05) when compared to that of the basal (0%) diet. Similarly, the supplementation of glycerin levels showed no significant influence (*p* > 0.05) on the relative GIT organ weights, carcass yield, or nutrient metabolism. Thus, we concluded that glycerin may be included in the broilers’ diets in rations of up to 15%.

## 1. Introduction

**Figure 1:**
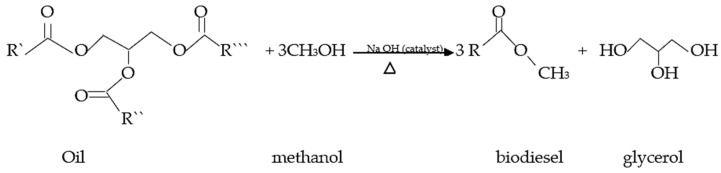
Transesterification reaction of oil, illustrating the resulting molecules: biodiesel and glycerol. Source: Adapted by the author based on [2].

Glycerin resulting from biodiesel production can be used to replace part of the cereal grains in the animal diet, as an alternative source of energy for farm animals in biofuel-producing countries [1]. Biodiesel is a product resulting from the transesterification reactions of triglycerides, both of animal and vegetable origin. In this reaction, alkaline catalysts are used. The transesterification process is an alcoholysis reaction where the final products are three molecules of methyl or ethyl esters of fatty acids (biodiesel) and one molecule of glycerol or glycerin [2], as can be observed in Figure 1. Glycerin applies to purified commercial products, usually containing at least 95% glycerol. Its composition influences characteristics such as color, odor, and impurities. When purified, glycerin presents a glycerol content of up to 99.5%.

In the chemical constitution of glycerin, there is a significant amount of glycerol, a molecular component that is easily absorbed through the intestinal wall and has a low molecular weight, being absorbed instantly without the mediation of micelles. After absorption, it is transported in the blood to the liver via aquaglyceroporins. In the liver, most of it is metabolized. Reabsorption and metabolization also occurs in the kidneys to avoid loss in the urine [3,4].

Glycerin contains glycerol, a major component synthesized from fatty acids known as triacylglycerols and the source of chemically storable energy as it is a precursor for the metabolization of glycerol-3-phosphate, a typical molecule of the glycolytic pathway. That is where glycerol is then converted into glucose, participating in the Krebs cycle or via gluconeogenesis, impacting the energy supply to the animal’s body [5].

Therefore, glycerin plays a fundamental role in animal and human energy metabolism because the glycerol present in their composition is an important structural component of triglycerides and phospholipids. The glycerol provided through the inclusion of glycerin in the diet is one of the compounds that are metabolized in the liver, which can be used in the lipogenesis or gluconeogenesis pathways. In a situation of excess energy, glycerol acts as a precursor for the synthesis of triglycerides, while in a phase of energy shortage, glycerol is used to provide carbon skeletons for gluconeogenesis or to produce energy through glycolysis and the Krebs cycle [6,7].

Glycerin is recognized as safe for use in animal feed [3]. However, it has a variable composition, affecting the nutritional value due to the characteristics of the compound used in the biodiesel production process that adopts the process of transesterification, resulting from the action of methanol on the vegetable oil. Salt is then added and this salt, when in excess, is a special concern for the use of glycerin in poultry production [8].

Up to 8% of sodium or potassium salts can be present in glycerin. In this case of chicken feed, as excretion increases, the quality of the litter worsens. As such, a diet formulated with 15% crude glycerin can present with around 1.26% sodium, where the amount of sodium in a basal ration is usually lower, at around 0.3% sodium [9].

The production of biodiesel in Brazil, due to the type of processing, can lead to glycerin containing around 7% salt. And thus, when adding glycerin into the diet, there may be an excess in relation to the nutritional needs of broiler chickens, as according to the Brazilian Table of Nutritional Requirements, approximately 0.183% to 0.227% of sodium is indicated in the feed. The chemical quality of Brazilian glycerin originating from biodiesel processing is a factor of concern for nutritionists when formulating glycerin-containing diets for animals [9,10].

The concern about excess salt in the diet of chickens arises due to the possibility of resulting in metabolic imbalance. For example, salt stimulates water consumption, and increased water intake results in higher litter moisture, which, in turn, increases ammonia excretion. This can cause respiratory problems in the birds, affecting their health and consequently the performance of the chicken [8,11].

In animal nutrition, studies have shown the benefits of glycerol in the diets of production animals [12]. When evaluating the ingestive behavior of sheep supplemented with increasing levels of glycerol, it was observed that the inclusion of up to 12% would not significantly interfere with nutrient intake, feed efficiency, or even dry matter and ingested fiber rumination.

So, the aim is to use glycerin appropriately, considering its chemical composition, with the purpose of it being an ingredient that contributes to the animals’ energy needs and thus being able to replace food sources such as cereals [13].

Studies on the levels of glycerol in the diet of non-ruminant animals, for example, pigs have been conducted, where the inclusion of 2.76% crude glycerin was found to have a significant effect on increasing weight gain in piglets during the nursery phase [14]. The use of glycerin in poultry diets was reported as early as 1976 [15]. They used glycerin inclusion levels of between 5% and 10% [16,17]. Taken together, these experiments indicate that glycerin can be added to broiler diets without adverse effects. However, gaps in knowledge remain.

The effects of including glycerin into the diet of chickens is still unknown, including their levels in the chickens’ diet, as well as the effects on their development during production in relation to nutrient metabolization and on weight gain during their rapid growth. Therefore, the present study was carried out to evaluate the effect of 0%, 5%, 10%, and 15% glycerin on performance, protein metabolism, fat and dry matter, carcass yield, and the relative weight of organs from the gastrointestinal tract of broiler chickens. This would provide a possible indication of glycerin produced from processed biodiesel in Brazil as a suitable ingredient in the feed of non-ruminant animals, such as broiler chickens, raised in the country to feed people all around the world.

## 2. Materials and Methods

### 2.1. Birds and Housing

The present study was conducted in the Poultry Research Farm of Federal Institute Goiano (Goiás, Brazil). The care and handling of all experimental birds were performed under protocols approved by the Institutional Animal Care and Use Committee of Federal Institute Goiano, protocol number 1329210317.

A total of 400 one-day-old male Cobb^®^ chicks were used, weighed individually and randomly distributed into 20 boxes measuring 1 m × 2 m × 0.60 m. With 20 chickens in each treatment group, they were subjected to one of the four treatments and had five replications of each test diet. The chicks had an initial weight of 40 ± 0.26 g, housed in a two-meter square stall, and the floor was covered with dry grass and clippings. Food and water were provided ad libitum throughout the experimental period and each box had a trough-type feeder and waterer. The chickens were transferred to metabolic cages at 35 days of age and after adaptation, the size of the cages was 0.90 m × 0.60 m × 0.40 m. The feed and excreta samples were collected for 5 days from the 34th day. A light program was adopted for 24 h, with natural and artificial light, while temperature and humidity were measured using a thermo hygrometer. The morning and afternoon averages were recorded, as can be seen in Table 1. Feed and clean drinking water were provided ad libitum throughout this experimental period. In Table 1, the data of temperature and relative humidity observed during the experiment are presented.

### 2.2. Experimental Design and Dietary Treatments

The chickens were distributed in a completely randomized design with four treatment groups and five replicates each. Therefore, each group had 20 chickens each, representing the four treatments and five replications of each test diet. The treatments consisted of four levels of dietary supplementation of gross energy glycerin (basal diet, 5, 10, and 15%).

The glycerin used in this experiment was obtained from a local industry and contained the following by composition analyses: 80.8% of glycerol, 111.0 mg/kg of methanol, and 4.015 kcal AME/kg of gross energy.

The experimental diets were formulated to have similar nutritional value according to [10]. The quantity of ingredients used to formulate the chicken diet was calculated according to the nutritional chemical analysis of each one before starting the experiment. A program was used to calculate the amount of each ingredient according to the animals’ nutritional requirements at each stage of chicken development. The initial diets are described in Table 2 below, where their nutritional composition is presented according to the needs of animals aged 1–21 days.

Table 3 contains the nutritional composition of the diet according to the needs of the animals in the growth phase, which covers the period from 22 to 35 days of age.

Table 4 presents the nutritional composition of the diets used to meet the nutritional needs required in the period from 36 to 42 days of life.

### 2.3. Measurements

#### 2.3.1. Performance Traits 

Body weight was recorded by weighing the birds per treatment group on days 7, 14, 21, and 42. Feed consumption for each treatment was measured by measuring feed residue on the same days that the chickens were weighed. The consumption was then estimated by the average of the five replicates representing the four treatments/test diets. The feed conversion rate (FCR, %) was calculated as the feed intake divided by the body weight gain (BWG, g) of the chickens. All performance parameters were calculated cumulatively according to the number of birds distributed (with 20 chickens in each of the four studied treatment groups), while considering the average of the five repetitions, to obtain the results for discussion from each evaluation period (7, 14, 21, and 42 days).

#### 2.3.2. Metabolism Analysis

For this analysis, five birds from each replicate were transferred to metabolic cages at 35 days of age. After adaptation (3 days), feed and excreta samples were collected for 3 days from the 41st day. The excreta collected on trays were identified and stored in plastic bags in a freezer until laboratory analysis. To mark the beginning and end of collection, 1% ferric oxide was used as an indicator.

Diets and excreta samples were stored at −20 °C in preparation for further analyses. After a period of storage, the chicken diets and excreta were thawed, weighed, homogenized, and pre-dried in a forced circulation oven at 55 °C. The excreta aliquots were crushed, and the analysis of dry matter (DM), crude protein (CP) and ether extract (EE) were performed following the methodology of Silva and Queiroz [13] at Federal Institute Goiano.

#### 2.3.3. Carcass Yield 

At the end of the experiment (42 d), five broiler chickens from each repetition were chosen from the group of twenty individuals, according to the average weight and subjected to 8 h of food restriction. All were males as only male chickens were raised in this study since they represent the category of broilers for meat production. Furthermore, in Brazil, meat production from chickens commonly involves the rearing of only males, due to the better conformation of the commercial cuts demanded by the local consumer. After food restriction, the selected chickens were sacrificed by cervical dislocation and bled immediately. Subsequently, they were scalded in hot water (55 °C) in an electric bath for two minutes, plucked, and eviscerated manually. The breast, thigh, and abdominal fat were manually removed from the carcasses and weighed, then the percentages relative to body weight were calculated.

Formula used to calculate the carcass yield of slaughtered animals:

= (sample weight/live weight) × 100

The samples are represented by the collected cuts, that is, portions of the body of the broiler chickens that were evaluated.

#### 2.3.4. Organ Weight, Blood Sampling, and Serum Biochemistry

On days 7, 14, 21, and 42, five broilers from each treatment were euthanized to assess the relative weights (%) of the GIT organs. The gizzard (Giz, %), pancreas (Pan, %), liver (Liv, %), small intestine (SI, %) (from the end of the muscular stomach to Meckel’s diverticulum) and large intestine (LI, %) (from Meckel’s diverticulum to rectum, including ceca) were removed and weighed following the methods described by Stringhini [18]. The relative weights of the GIT organs were calculated according to the following formula: organ relative weight % = (organ weight/body weight) × 100.

To assess the serum biochemical profile, the blood from the euthanized animals was collected by cardiac puncture from the five broilers per treatment at day 42. Blood samples were processed according to the method described by Minafra [19]. The serum levels of Calcium (Ca, mg/mL), phosphorus (P, mmol/L), chlorine (Cl, mmol/L), sodium (Na, mmol/L), potassium (K, mmol/L), total protein (Prot, g/dL), cholesterol (Chol, mg/dL) and triglyceride (Trig, mg/dL) were analyzed. Blood serum analyses were performed in the Laboratory of Animal Biochemistry and Metabolism (Federal Institute Goiano). All analyses were determined in triplicate and were measured spectrophotometrically (Spectrum^®^—Celer, Belo Horizonte, MG, Brazil) using a commercial kit protocol (Doles^®^, Goiania, GO, Brazil).

### 2.4. Statistical Analysis

Performance data, carcass yield, relative weights of GIT organs and nutrient metabolism were the serum biochemical parameters that were subjected to an analysis of variance (ANOVA), while the treatment means were evaluated by Tukey’s test at 5% significance per the SISVAR^®^ statistical software (5.6), 2019 [20].

## 3. Results

The results of the effects of glycerin supplementation on performance in broiler chickens are shown in Table 5. No significant differences in BWG (g), FI, and FCR (g/g) were observed (*p* > 0.05) across the increasing glycerin levels in the diet throughout experimental period.

The diets containing 5, 10, or 15% level of inclusion of GLY had statistically similar digestibility of crude protein and fat when compared to the basal diet (*p* > 0.05, Table 6).

No significant differences were observed in the yield of carcass, breast, thigh, drumstick, and abdominal fat among the different treatments (*p* > 0.05, Table 7).

The relative weights of the GIT organs are shown in Table 8. No differences were found in the organ weights among broilers fed with the 0, 5, 10, or 15% glycerin diets (*p* > 0.05).

The serum levels of Chol and Trig were not affected (*p* > 0.05) by the increasing levels of glycerin in the diet (Table 9). Levels of Calcium (Ca), phosphorus (P), chlorine (Cl), sodium (Na), potassium (K), total protein (Prot), cholesterol (Chol) and triglyceride (Trig) were also not affected by the increase in glycerin in the diets.

## 4. Discussion

The increases in feed costs and raw material prices have become necessary to formulate the most economical and balanced feeds with the available co-products. Our study demonstrated that the inclusion of 5, 10, or 15% glycerin in broiler diets does not promote adverse effects on performance, providing an indication that glycerin may be a viable alternative to partially replace other ingredients for energy.

As birds regulate their feed consumption depending on the level of AME in the diet [21], the glycerol present in glycerin can be used in poultry nutrition as it has a high energy value [1,22]. Glycerin also contains variable amounts of substances, which could limit its use as an ingredient in animal diets as it presents variability in its components and consequently diversity in the AME values. The AME content for glycerin used in this research was 4015 kcal. As the experimental diets assumed the same AME at different ages, we found that the FI in the basal diet was similar to the increasing levels of the glycerin dietary groups. On the other hand, [23] showed that during the development of the pre-initial phase (1–10 d), there was higher FI in the groups fed with levels of 6, 9, and 12% of crude GLY compared to the control, which is different from Romano’s findings [17] which observed higher AI in broilers aged 8 to 12 days fed with 2.5 and 7.5% of crude glycerin than in those fed with 10%. However, the same study indicated that as the birds aged, there were no differences in feed consumption.

In the studies carried out on the feeding of broiler chickens that included up to 10% of crude glycerin in the feed in the pre-initial phase, they observed that it was favorable to the performance of the chickens in the initial phase; however, as the animals grew, the 5% level positively affected the development of animals instead [24].

Fontinele et al. [25] and Sehu et al. [26] suggested that a 10% concentration is the optimal level of inclusion of crude glycerin in the diet to have beneficial effects on broiler performance. Despite this, [27] reported that the addition of 10% glycerin to the feed resulted in feed flow problems that may have caused a reduction in FI and consequently in BW. In this study, the inclusion of 5, 10, or 15% resulted in similar body weight gain between the groups. Furthermore, no feed consumption problems were observed, even in the 15% glycerin treatment, indicating to us that glycerin can be included at levels of up to 15% for broiler chickens without any negative effects on performance parameters.

Glycerin can affect the physical structure of the feed in relation to texture and consistency, encouraging consumption by animals. Despite the increase in consumption, to date, no interference was observed in weight gain and carcass quality. However, in meat quails, there was an increase in abdominal fat, especially when fed with glycerin of a vegetable or mixed origin, a fact not observed in quails fed with glycerin (semi-purified using a cutting machine) from the manufacture of biodiesel [28].

In the case of semi-purified glycerin at a level of up to 16%, it did not negatively interfere with feed consumption and weight gain, despite the worsening feed conversion as the level of the diet increased, a fact that may be due to the sodium levels in the diet that can lead to increased moisture in excreta and thus interfere with energy metabolism [29,30].

As a performance enhancer for broiler chickens, it is reported in the literature that glycerin levels of 5% and 10% can positively influence animal performance, with an emphasis on increasing feed conversion. Furthermore, it is important to highlight the possibility of reducing daily weight gain when glycerin is being produced. This is because the metabolites can be lost in the urine when in excess and thus the ability to increase energy is lost. Consequently, the potential for weight gain is reduced, especially in the final phase of production where the chicken’s organs are mature and are capable of recycling nutrients or excreting excess, when necessary, as is the case with the kidneys [31,32].

Considering the lack of effect, this may be due to the type of glycerin, raw, mixed, or semi-purified, as they do not present significant differences in the effect on chicken performance. As already published in a study on the use of different types of glycerin in chickens aged of up to 21 days, that is, in the structural development phase of the animals’ organism, despite the need for more nutrients at this stage, the feed consumption or weight gain of the animals remains without interference from the inclusion of glycerin [33].

Glycerol is known as an easily digestible source of energy [34,35]. The bioavailability of glycerol in broiler chickens is believed to be greater than other energy sources except oils and glucose. Studies have reported the impact of glicerin inclusion on energy digestibility, but only a few of them have shown effects on protein and fat digestibility.

**Figure 2:**
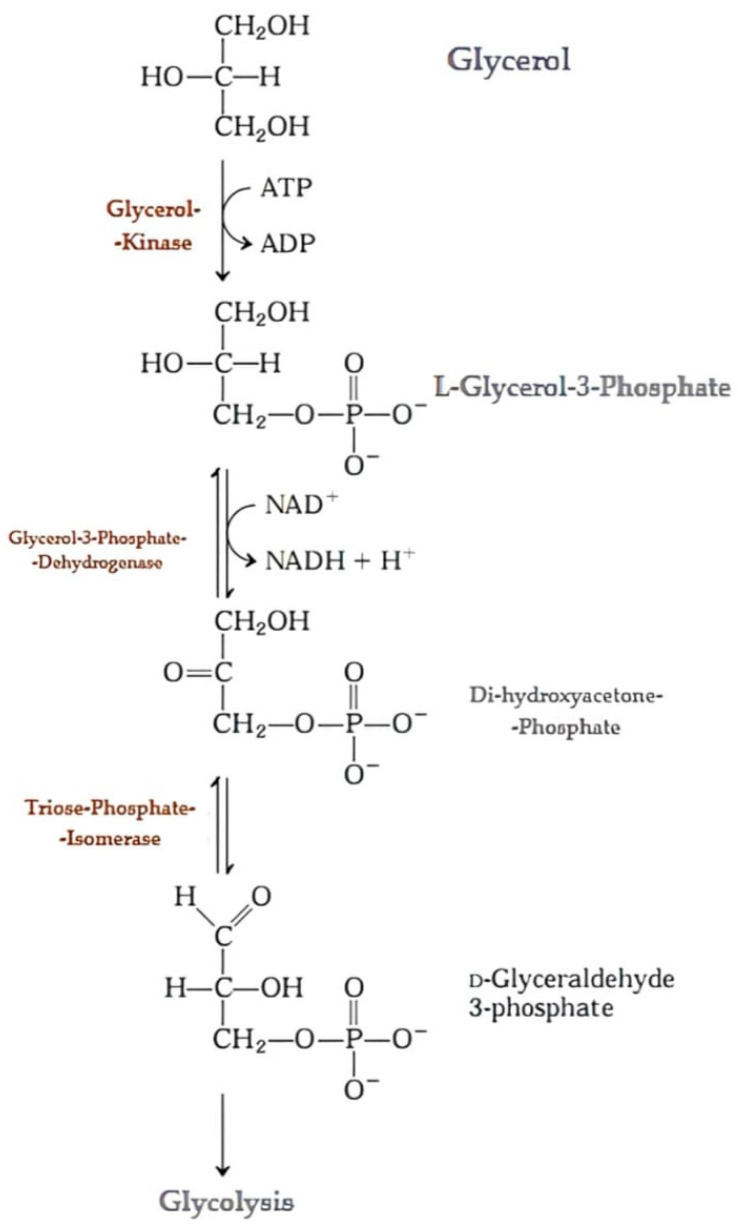
Metabolism of glycerol to enter the glycolytic pathway. Source: Adapted from [36].

In glycerol metabolism, we have this molecule as an energy source because it undergoes lipase action and is then phosphorylated by glycerol kinase. It is thus oxidized until it forms dihydroxyacetone phosphate, a molecule which, in turn, is oxidized and enters the glycolytic pathway to form ATP (Adenosine triphosphate). This reaction can be observed in Figure 2. The glyceraldehyde phosphate formed is oxidatively phosphorylated, producing NADH and ATP, resulting in 1,3-bisphosphoglycerate, which is then dehydrated to form phosphoenolpyruvate, yielding more ATP. Finally, the pyruvate molecule can be fermented to produce ethanol and lactate or can also enter the glycolytic pathway. In glycolysis, pyruvate releases carbon and becomes Acetyl-CoA, which enters the citric acid cycle [36].

In addition to the energy that can be produced in the animal through the metabolism of glycerol, the pyruvate obtained can have its excess stored in the form of fat. That is, when pyruvate in the glycolytic pathway is in the form of Acetyl-CoA, a molecule resulting from the transformation of excess pyruvate acted upon by the enzyme acetyl-CoA carboxylase, this molecule (Acetyl-CoA) then participates in the fatty acid synthesis pathway. Upon synthesis, the molecule converts into triacylglycerols, which are deposited in adipocytes. This reaction is reversible for gluconeogenesis when low glucose levels occur [36].

Therefore, the inclusion of fat in the diet increased the digestion retention time in chickens, which subsequently increased the use of energy, protein, and fat [37,38]. Noting that the inclusion levels influence bird health and indicate that glycerin can be added up to levels of 5% to broiler diets without an effect on performance [39]; this observation led to the hypothesis that a 10% increase in dietary glycerin may influence nutrient utilization from protein and fat in diets, since the chemical components in glycerin are like the enzymatic hydrolytic products of dietary fat metabolism. However, we found that the addition of 15% glycerin to diets did not influence the protein and fat digestibility of broiler chickens.

Our data indicated that chickens fed with up to 15% glycerol experienced a slight numerical increase in carcass, breast, thigh, and drumstick yield, but there were no statistically significant differences between treatments. Furthermore, ref. [27] stated that the inclusion of 10% glycerin in the chicken diet can cause a reduction in the yield of whole carcasses, without affecting the yield of cuts such as breasts and thighs.

Wattanachant et al. [40] provided 2.5%, 5%, 7.5%, and 10% glycerin in pellets ad libitum to one-day-old broilers until 42 days of age. No significant difference was found in live weight variation and feed intake between those receiving diets with and without glycerin supplementation. However, low growth performance and carcass yield were observed when the glycerin levels reached 10%. Similarly, ref. [23] observed no difference in the rate of fat deposition when the chickens were fed with glycerin levels of 3%, 9%, 12%, or 15%. However, our results disagree with [27], who reported that birds fed with 2.5 or 5% glycerin had significantly higher breast yield than the control diet. Available data suggest that 15% GLY supplementation is not harmful to the carcass, breast, thigh, and drumstick yield and does not lead to the deposition of abdominal fat.

There was no effect of adding 5, 10, or 15% glycerin to the diet on the relative weights of the GIT organs of broiler chickens when compared to the control group. This result agreed with that of Topal and Ozdogan [35], who concluded that the inclusion of glycerol in diets did not affect the weight of the internal organs of male and female broilers, except the heart weight of males in the 8% glycerin group. An excess of glycerol in the diet can induce anatomical, physiological, and biochemical adaptation of organs [37].

There is considerable concern about the use of glycerin as a dietary approach because the enzymatic capacity for glycerol metabolism is limited. When there is a high level of glycerol in the diet, consumption can exceed the metabolic capacity, consequently increasing blood levels [39]. Glycerol is an important structural component of triglycerides and phospholipids. Dietary glycerol is metabolized in the liver, which can be used in the lipogenesis or gluconeogenesis pathways. In a situation of excess energy, glycerol acts as a precursor for the synthesis of triglycerides, while in a situation of energy scarcity, it is used to provide carbon skeletons for gluconeogenesis or to produce energy through glycolysis and the energy cycle—Krebs [41,42].

Our results show that fat parameters at levels of 5, 10, or 15% glycerin in the diet remained similar to those in the control group, clearly indicating that 15% glycerol can be used without negative effects on fat metabolism. In contrast, [43] found that the estimated plasma glycerol level increased 2 h after feeding with the 5% glycerin supplementation, from 0.65 (control) to 4.36 mmol/l.

According to [44], glycerol can affect the hydration status and nutrient metabolism, or both. Water consumption measurements were not performed in the present experiment; however, serum electrolytes, Ca/P ratio, and metabolism parameters [45,46] were performed in another study to determine whether the addition of increased glycerin to diets could disrupt nutrient balance. Another concern about the use of glycerol is related to the variety of residual levels of methanol, sodium, potassium, and moisture that can also lead to electrolyte imbalance. Our results show that the addition of glycerin levels to broiler diets does not negatively influence serum biochemical parameters.

## 5. Conclusions

Our results indicate that glycerin from the processing of biodiesel of plant origin can be included in the diets of broiler chickens in feed of up to a level of 15% as it does not present negative effects for breeding. This is a feed of interest while corn is on the rise, due to the low supply and competition of the grain as food for people, and the fact that glycerin is in great supply, as well as the possibility of using it on a large scale for feeding animals as there is no high food competition for this ingredient.

This study points out that to expand the use of glycerin as an energy ingredient in the feed of production animals, it is necessary to invest in improvements in the standardization of the chemical composition of glycerin to make this food efficient, as well as to rethink the price. We therefore recommend future research for the economic viability and nutritional value of this product.

## Figures and Tables

**Table 1:** Temperature and humidity during the rearing of the chickens in the pre-starter (1–7 days), starter (8–14 days), growth (15–21 days), and finisher (22–42 days) phases.

Rearing Phase	Average Temperature		Average Humidity	
	Maximum °C	Minimum °C	Maximum %	Minimum %
Pre-starter	31.05	18.22	69.00	56.00
Starter	30.83	17.21	69.52	50.00
Growth	29.77	16.54	65.00	57.00
Finisher	27.83	16.00	64.00	58.87

**Table 2:** Ingredients and the calculated nutritional composition of starter diets (1–21 d) according to increasing levels of glycerin.

Ingredients	Glycerin Inclusion (%)			
	0	5	10	15
Corn	59.30	53.86	48.06	42.27
Soybean meal	34.70	35.40	36.52	37.65
Soybean oil	2.44	2.01	1.69	0.37
Dicalcium phosphate	1.81	1.83	1.84	1.85
Limestone	0.83	0.82	0.80	0.79
Common salt ^1^	0.44	0.44	0.45	0.45
DL-methionine	0.27	0.28	0.29	0.29
L-lysine HCL	0.09	0.24	0.22	0.20
Mineral premix ^2^	0.04	0.04	0.04	0.04
Vitamin premix ^3^	0.04	0.04	0.04	0.04
Antioxidant	0.01	0.01	0.01	0.01
Glycerin	0.00	5.00	10.00	15.00
**Nutritional Composition**				
AMEn (Kcal/kg)	3.050	3.050	3.050	3.050
Crude protein (%)	23.00	23.00	23.00	23.00
Calcium (%)	0.87	0.87	0.87	0.87
Phosphorus (%)	0.41	0.41	0.41	0.41
Lysine (%)	1.25	1.25	1.25	1.25
Met. + cyst. (%)	0.92	0.92	0.92	0.92
Methionine (%)	0.51	0.51	0.51	0.51
Sodium (%)	0.21	0.21	0.21	0.21

^1^ The composition of sodium chloride includes approximately 39% sodium, 60% chlorine, and 1% iodine. ^2^ Contained per kg of the product: Manganese 150 g; Zinc 100 g; Iron 100 g; Copper 16 g; Iodine 1.5 g. ^3^ Contained per kg of the product: Folic Acid 1.600 mg; Pantothenic Acid 29.000 mg; Biotin 60 mg; Butylated hydroxytoluene (BHT) 5.000 mg; Niacin 7.000 mg; Vitamin A 20.000,000 mg; Vitamin B1 3.000 mg; Vitamin E 40.500 UI; Vitamin B12 27.000 mg; Vitamin B2 12.000 mg; Vitamin B6 6.000 mg; Vitamin D3 5.000 UI; Vitamin K3 4.800 mg.

**Table 3:** Ingredients and the calculated nutritional composition of grower diets (22–35 d) according to increasing levels of glycerin.

Ingredients	Glycerin Inclusion (%)			
	0	5	10	15
Corn	62.53	56.74	50.94	45.15
Soybean meal	30.68	31.82	32.93	34.06
Soybean oil	3.30	2.98	2.66	2.34
Dicalcium phosphate	1.67	1.68	1.70	1.71
Limestone	0.79	0.78	0.76	0.75
Common salt ^1^	0.42	0.42	0.42	0.43
DL-methionine	0.25	0.26	0.26	0.27
L-lysine HCL	0.25	0.23	0.21	0.19
Mineral premix ^2^	0.03	0.03	0.03	0.03
Vitamin premix ^3^	0.03	0.03	0.03	0.03
Antioxidant	0.01	0.01	0.01	0.01
Glycerin	0.00	5.00	10.00	15.00
**Nutritional Composition**				
AMEn (Kcal/kg)	3.150	3.150	3.150	3.150
Crude protein (%)	21.00	21.00	21.00	21.00
Calcium (%)	0.75	0.75	0.75	0.75
Phosphorus (%)	0.37	0.37	0.37	0.37
Lysine (%)	1.12	1.12	1.12	1.12
Met. + cyst. (%)	0.91	0.91	0.91	0.91
Methionine (%)	0.51	0.51	0.51	0.51
Sodium (%)	0.20	0.20	0.20	0.20

^1^ The composition of sodium chloride includes approximately 39% sodium and 60% chlorine and 1% iodine. ^2^ Contained per kg of the product: Manganese 150 g; Zinc 100 g; Iron 100 g; Copper 16 g; Iodine 1.5 g. ^3^ Contained per kg of the product: Folic Acid 1.600 mg; Pantothenic Acid 29.000 mg; Biotin 60 mg; Butylated hydroxytoluene (BHT) 5.000 mg; Niacin 7.000 mg; Vitamin A 20.000,000 mg; Vitamin B1 3.000 mg; Vitamin E 40.500 UI; Vitamin B12 27.000 mg; Vitamin B2 12.000 mg; Vitamin B6 6.000 mg; Vitamin D3 5.000 UI; Vitamin K3 4.800 mg.

**Table 4:** Ingredients and the calculated nutritional composition of finisher diets (35–42 d) according to increasing levels of glycerin.

Ingredients	Glycerin Inclusion (%)			
	0	5	10	15
Corn	66.81	61.02	55.22	49.42
Soybean meal	26.65	27.77	28.90	30.03
Soybean oil	3.23	2.91	2.59	2.27
Dicalcium phosphate	1.25	1.57	1.55	1.56
Limestone	0.76	0.74	0.73	0.71
Common salt ^1^	0.40	0.40	0.40	0.41
DL-methionine	0.24	0.25	0.26	0.27
L-lysine HCL	0.31	0.29	0.27	0.25
Mineral premix ^2^	0.02	0.02	0.02	0.02
Vitamin premix ^3^	0.02	0.01	0.02	0.02
Antioxidant	0.01	0.01	0.01	0.01
Glycerin	0.00	5.00	10.00	15.00
**Nutritional Composition**				
AMEn (Kcal/kg)	3.200	3.200	3.200	3.200
Crude protein (%)	19.00	19.00	19.00	19.00
Calcium (%)	0.65	0.65	0.65	0.65
Phosphorus (%)	0.29	0.29	0.29	0.29
Lysine (%)	1.01	1.01	1.01	1.01
Met. + cyst. (%)	0.75	0.75	0.75	0.75
Methionine (%)	0.41	0.41	0.41	0.41
Sodium (%)	0.19	0.19	0.19	0.19

^1^ The composition of sodium chloride includes approximately 39% sodium and 60% chlorine and 1% iodine. ^2^ Contained per kg of the product: Manganese 150 g; Zinc 100 g; Iron 100 g; Copper 16 g; Iodine 1.5 g. ^3^ Contained per kg of the product: Folic Acid 1.600 mg; Pantothenic Acid 29.000 mg; Biotin 60 mg; Butylated hydroxytoluene (BHT) 5.000 mg; Niacin 7.000 mg; Vitamin A 20.000,000 mg; Vitamin B1 3.000 mg; Vitamin E 40.500 UI; Vitamin B12 27.000 mg; Vitamin B2 12.000 mg; Vitamin B6 6.000 mg; Vitamin D3 5.000 UI; Vitamin K3 4.800 mg.

**Table 5:** Body weight gain (BWG), feed intake (FI), and feed conversion ratio (FCR) in broilers fed with increasing levels of glycerin throughout experimental period.

Treatments	BWG (g)	FI (g)	FCR (g/g)
	1–7 d		
0%	119.00	135.00	1.131
5%	120.50	133.00	1.100
10%	119.00	132.00	1.112
15%	120.00	135.00	1.133
CV%	2.98	3.66	4.87
*p* value	0.085	0.091	0.097
SEM	0.99	0.98	0.050
**Treatments**	**1–14 d**		
0%	430.54	509.75	1.180
5%	420.04	503.08	1.201
10%	409.45	476.93	1.161
15%	418.75	498.22	1.193
CV%	4.53	5.23	6.15
*p* value	0.407	0.710	0.218
SEM	0.93	1.46	0.04
**Treatments**	**1–21 d**		
0%	880.00	1120.00	1.270
5%	810.00	1060.00	1.310
10%	800.00	1080.00	1.351
15%	860.00	1100.00	1.282
CV%	4.53	4.81	5.10
*p* value	0.402	0.574	0.328
SEM	0.93	1.46	2.45
**Treatments**	**1–42 d**		
0%	2560.00	4100.00	1.601
5%	5570.00	4150.00	1.610
10%	2540.00	4010.00	1.582
15%	2560.00	4130.00	1.611
CV%	4.81	6.29	5.27
*p* value	0.575	0.681	0.159
SEM	1.01	1.58	0.71

SEM: Standard error of mean; CV: Coefficient of variation.

**Table 6:** Metabolism coefficients of crude protein, ether extract, and dry matter in broilers fed with increasing levels of glycerin in phase finisher.

Treatments	Crude Protein	Ether Extract	Dry Matter
0%	66.01	80.25	80.12
5%	65.99	80.26	80.00
10%	66.02	80.61	80.71
15%	65.97	80.30	80.52
CV%	3.35	4.69	4.16
*p* value	0.127	0.095	0.087
SEM	0.0003	0.0021	0.0019

SEM: Standard error of mean; CV: Coefficient of variation.

**Table 7:** Carcass yield of broilers of broilers at 42 days of age, fed with increasing levels of glycerin.

Treatments	Average weight of the Chickens (g)	Carcass (%)	Breast (%)	Thigh (%)	Drum Stick (%)	Abdominal Fat (%)
0%	2513	76.33	29.80	11.89	10.89	1.46
5%	2580	78.89	30.04	12.99	12.74	1.49
10%	2585	77.32	29.97	11.98	10.74	1.40
15%	2583	78.95	30.45	13.39	11.79	1.48
CV%	2.34	3.28	7.05	6.35	5.57	15.63
*p* value	0.091	0.180	0.166	0.152	0.155	0.150
SEM	0.20	0.26	0.36	0.19	0.42	0.18

SEM: Standard error of mean; CV: Coefficient of variation.

**Table 8:** Relative weights of gastrointestinal tract organs in broilers, fed with increasing levels of glycerin.

Treatments	Average Weight of the Chickens (g)	LIV (%)	GIZ (%)	PAN (%)	LI(%)	SI (%)
		**7 days**				
0%	159.48	4.25	8.06	0.51	1.41	6.00
5%	159.70	4.83	8.21	0.49	1.57	6.38
10%	161.67	4.70	8.55	0.50	1.48	6.51
15%	154.80	4.67	8.03	0.53	1.57	6.43
CV %	6.26	12.75	11.99	12.73	9.56	13.91
*p* value	0.533	0.181	0.155	0.224	0.158	0.148
SEM	0.11	0.20	0.33	0.13	0.21	0.44
**Treatments**		**14 days**				
0%	400.0	2.69	4.53	0.34	1.13	4.38
5%	428.0	3.05	5.06	0.32	1.03	5.08
10%	394.0	2.99	5.41	0.36	1.15	4.91
15%	406.8	3.04	4.96	0.34	1.17	4.98
CV %	9.07	7.87	8.39	9.17	11.79	10.15
*p* value	0.546	0.145	0.069	0.150	0.341	0.193
SEM	0.16	0.18	0.36	0.03	0.11	0.42
**Treatments**		**21 days**				
0%	936.0	2.47	3.76	0.26	0.80	3.56
5%	920.0	2.81	4.18	0.27	0.88	3.73
10%	920.0	2.57	4.03	0.27	0.98	3.90
15%	930.0	2.74	4.05	0.26	0.92	3.68
CV %	5.32	12.44	8.45	11.70	11.31	8.05
*p* value	0.128	0.355	0.297	0.153	0.068	0.395
SEM	0.34	0.16	0.32	0.03	0.11	0.29
**Treatments**		**42 days**				
0%	2513	1.59	1.88	0.16	0.80	2.70
5%	2580	1.61	1.81	0.15	0.77	2.70
10%	2585	1.75	1.80	0.16	0.77	2.71
15%	2583	1.79	1.80	0.16	0.79	2.71
CV %	2.34	8.43	8.88	6.98	11.47	8.66
*p* value	0.091	0.096	0.114	0.145	0.150	0.391
SEM	0.20	0.06	0.07	0.005	0.03	0.09

SEM: Standard error of mean; CV: Coefficient of variation.

**Table 9:** Serum biochemical parameters of broilers at 42 days of age, fed with increasing levels of glycerin.

Treatments	Parameters								
	Ca mg/mL	P mmol/L	Ca: P mmol/L	Cl mmol/L	Na mmol/L	K mmol/L	Prot g/dL	Chol mg/dL	Trig mg/dL
0%	10.09	5.08	2.01	81.92	120.18	6.05	4.68	108.51	231.85
5%	10.23	5.18	1.97	85.42	119.24	5.89	4.49	108.31	230.31
10%	10.09	5.31	1.89	87.96	120.89	5.99	4.47	108.40	232.12
15%	10.08	5.09	2.00	82.08	119.96	6.12	4.47	108.61	233.53
CV%	6.57	9.73	10.38	10.51	9.27	8.06	13.04	11.71	11.03
*p* value	0.155	0.159	0.151	0.139	0.126	0.943	0.155	0.150	0.153
SEM	0.19	0.20	0.13	0.25	1.12	0.87	0.98	0.87	0.69

SEM: Standard error of mean; CV: Coefficient of variation.

## Data Availability

Data are contained within the article.
